# EMS Responses for Pediatric Behavioral Health Emergencies in the United States: A 4-Year Descriptive Evaluation

**DOI:** 10.1017/S1049023X2300657X

**Published:** 2023-12

**Authors:** Lori L. Boland, Morgan K. Anderson, Jonathan R. Powell, Michael T. Patock, Ashish R. Panchal

**Affiliations:** 1. Clinical and Research Services, ImageTrend Inc., Lakeville, Minnesota USA; 2. Allina Health Emergency Medical Services, St. Paul, Minnesota USA; 3. Tulane University School of Public Health and Tropical Medicine, New Orleans, Louisiana USA; 4. Division of Epidemiology, The Ohio State University College of Public Health, Columbus, Ohio USA; 5. National Registry of Emergency Medical Technicians, Columbus, Ohio USA; 6. Department of Emergency Medicine, The Ohio State University Wexner Medical Center, Columbus, Ohio USA

**Keywords:** behavioral health, pediatric, prehospital

## Abstract

**Background:**

The occurrence of behavioral health emergencies (BHEs) in children is increasing in the United States, with patient presentations to Emergency Medical Services (EMS) behaving similarly. However, detailed evaluations of EMS encounters for pediatric BHEs at the national level have not been reported.

**Methods:**

This was a secondary analysis of a national convenience sample of EMS electronic patient care records (ePCRs) collected from January 1, 2018 through December 31, 2021. Inclusion criteria were all EMS activations documented as 9-1-1 responses involving patients < 18 years of age with a primary or secondary provider impression of a BHE. Patient demographics, incident characteristics, and clinical variables including administration of sedation medications, use of physical restraint, and transport status were examined overall and by calendar year.

**Results:**

A total of 1,079,406 pediatric EMS encounters were present in the dataset, of which 102,014 (9.5%) had behavioral health provider impressions. Just over one-half of BHEs occurred in females (56.2%), and 68.1% occurred in patients aged 14-17 years. Telecommunicators managing the 9-1-1 calls for these events reported non-BHE patient complaints in 34.7%. Patients were transported by EMS 68.9% of the time, while treatment and/or transport by EMS was refused in 12.5%. Prehospital clinicians administered sedation medications in 1.9% of encounters and applied physical restraints in 1.7%. Naloxone was administered for overdose rescue in 1.5% of encounters.

**Conclusion:**

Approximately one in ten pediatric EMS encounters occurring in the United States involve a BHE, and the majority of pediatric BHEs attended by EMS result in transport of the child. Use of sedation medications and physical restraints by prehospital clinicians in these events is rare. National EMS data from a variety of sources should continue to be examined to monitor trends in EMS encounters for BHEs in children.

## Introduction

Emergency Medical Services (EMS) are a critical entry point to the emergency health care system for many patients in the United States. This is particularly true for children suffering acute behavioral health emergencies (BHEs) who often require immediate transport to facilities with specialized pediatric behavioral health infrastructure for evaluation and possible hospitalization.^1,2^ Unfortunately, the occurrence of pediatric BHEs across the United States has been increasing. Data from the National Hospital Ambulatory Medical Care Survey (Centers for Disease Control and Prevention; Atlanta, Georgia USA) reveal that the proportion of emergency department (ED) visits among youth involving mental health conditions rose from 4.4% in 2001 to 7.2% in 2011,^3^ and continued to rise to 10.1% by 2015.^4^ Accompanying this rise are continued concerns about the inadequacies of the emergency health care system to address the needs of youth experiencing BHEs.^5,6^

As pediatric BHE volumes have increased, patient presentations to EMS have similarly increased. Approximately 26% of pediatric patients seen in EDs for BHEs between 2011 and 2015 arrived by ambulance.^4^ Studies using United States national EMS data have shown behavioral and psychiatric conditions accounted for approximately 15% of all 9-1-1 responses in children in 2016^7^ and that 7.3% of all 9-1-1 responses for BHEs in 2018 involved patients under the age of 18.^8^ However, detailed evaluations of EMS encounters involving pediatric BHEs at the national level have not been done, and prior studies have often excluded events that do not result in transport.^9–11^ With the rate of non-transport in pediatric EMS encounters in the United States approaching 30%,^12^ the care and management of pediatric BHE patients who are not transported requires further evaluation.

There are no national descriptions of the EMS care provided to children experiencing BHEs that illuminate how the escalating pediatric mental health crisis in the United States is manifesting in the prehospital care domain. In this study, the objective was to describe the prevalence, associated demographics, and prehospital care of pediatric patients presenting to EMS with BHEs in the United States from 2018 through 2021.

## Methods

This study was a secondary analysis of a large national convenience sample of EMS electronic patient care records (ePCRs) collected by agencies across the United States from January 1, 2018 through December 31, 2021. The study protocol was evaluated by a human research subjects review board (Sterling IRB [Atlanta, Georgia USA] #8700-MKAnderson) and was deemed exempt from review.

### Data Source

ImageTrend software solutions (ImageTrend; Lakeville, Minnesota USA) are commonly used by emergency responders across the United States for the electronic capture and reporting of operational and clinical data related to emergency response incidents. This evaluation utilized the Collaborate database maintained by ImageTrend, which is a de-identified, prehospital electronic health record dataset that currently comprises approximately 44 million EMS activations from 2,502 EMS agencies providing emergency medical response in 50 United States’ states and territories. The dataset is continually updated with new events forming a real-time data repository for industry-relevant surveillance, reporting, and research. The EMS agencies have the option to decline having their data become part of Collaborate. The dataset is compliant with the National EMS Information System (NEMSIS; Salt Lake City, Utah USA) Version 3 data standard,^13^ and thus reflects the most current universal standards for the collection and definition of prehospital patient encounter datapoints, including several hundred data validation rules.^14^ Data for this study were extracted from the proprietary Collaborate dataset using an SQL interface and filters reflecting the inclusion and exclusion criteria described below.

### Measurements

Patient demographics and clinical variables used in this analysis reflect EMS clinician documentation extracted from the ePCRs, thus leveraging NEMSIS data definitions. Patient demographics included sex and age. Several response characteristics were also analyzed, including location of patient encounter, the complaint recorded by the 9-1-1 telecommunicator, provider impressions indicated by the EMS clinician, patient disposition, and EMS interventions. Age is recorded as a continuous variable by prehospital clinicians, however, for purposes of analysis, age was evaluated using the following categories corresponding to United States education levels and reflecting previous definitions proposed by the National Institute of Child Health and Human Development (Rockville, Maryland USA):^15^ 0-4 years (early childhood), 5-10 years (elementary), 11-13 years (middle school), and 14-17 years (high school). Location of the patient encounter was categorized as residence (eg, private residence, apartment); school (eg, school, daycare); street; health care facility (eg, hospital, clinic); commercial setting (eg, restaurant/café, airport); public area/building; recreational area (eg, park, pool); or other using NEMSIS coding as previously described.^8^ Primary provider impression is the clinician’s impression of the primary problem or most significant condition that led to the patient care provided. A clinician’s secondary impression indicating a second, less-severe problem which may or may not be directly related to the primary impression can also be listed. Patient disposition was used to describe whether the patient was transported by EMS. Use of physical restraints and the administration of specific medications frequently used in the management of BHE patients were also examined. Medications of interest included sedation medications as well as naloxone, which is commonly used in cases of opioid overdose. In addition to patient demographics and clinical variables, an indicator of urbanicity where the event occurred was derived using the incident county when available in the ePCRs. Urbanicity was defined using the Rural-Urban Continuum Code (RUCC) assigned to the incident county based on 2013 United States census data.^16^ The RUCC scheme categorizes counties into three metropolitan and six non-metropolitan classifications, which were categorized as metro area (RUCC = 1-3), non-metro area (RUCC = 4-7), and rural (RUCC = 8-9) for purposes of analysis.

### Selection of EMS Events

Included in the analysis were all EMS activations documented as 9-1-1 responses involving patients < 18 years of age with a behavioral health provider impression occurring from January 1, 2018 through December 31, 2021. Behavioral health impressions were defined as International Classification of Diseases 10^th^ Revision (ICD-10) codes F01-F99, which represent mental, behavioral, and neurodevelopmental disorders. Events were excluded if they did not result in any patient contact or if the primary provider impression was missing.

### Analysis

The primary outcome was the overall frequency of EMS presentations involving BHEs in children. Descriptive statistics were calculated for the demographic, clinical, and incident characteristics of the total population of pediatric BHE events, as well as by calendar year. All analyses were conducted using Microsoft Power BI analytics software Version 2.112.603.0 (Microsoft Corporation; Redmond, Washington USA) and Microsoft Excel 2016.

## Results

**Figure 1.**
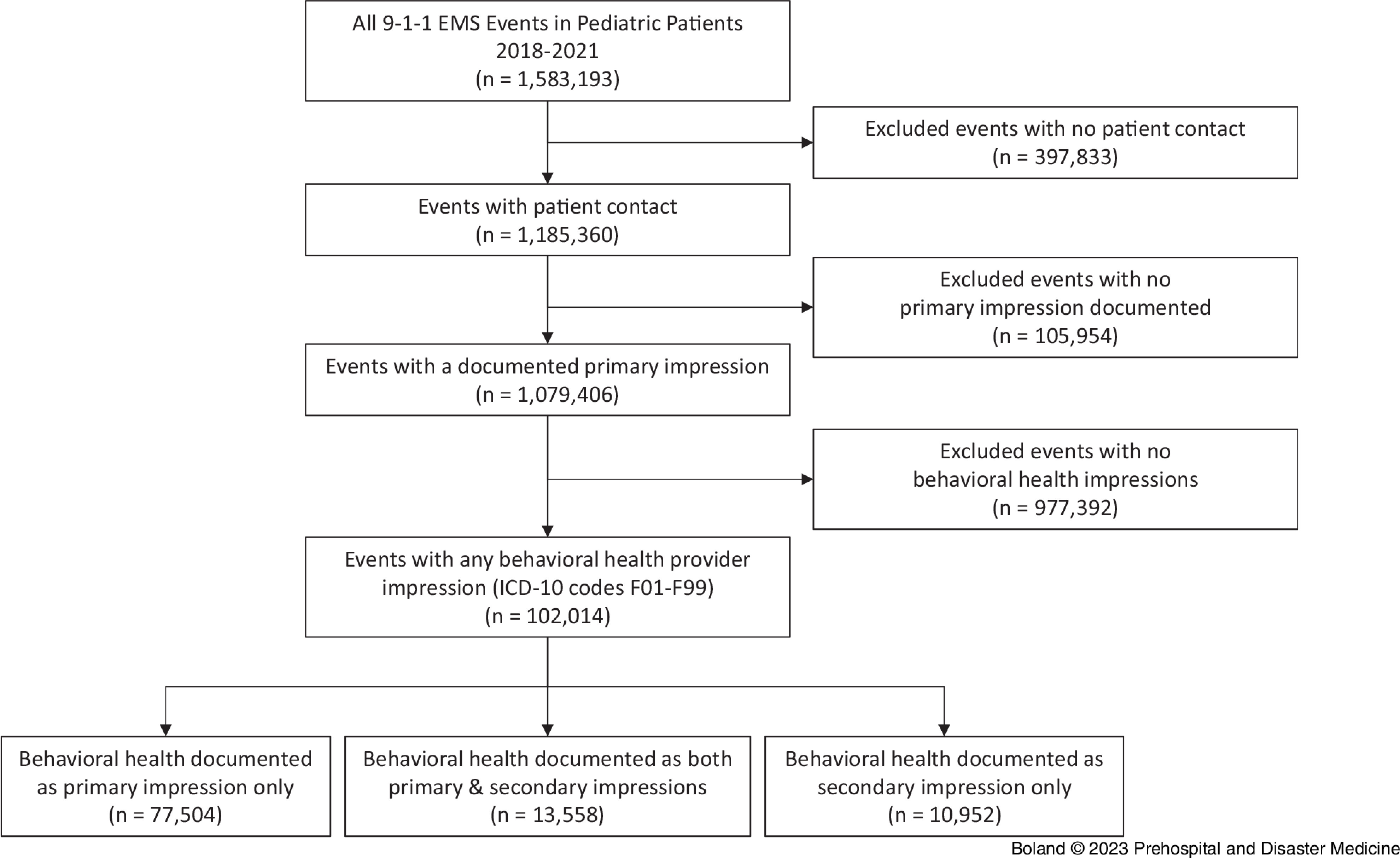
Flow Diagram of EMS Events Involving Pediatric Patients (<18 years) Who Had at Least One Behavioral Health Provider Impression Documented by EMS. Abbreviations: EMS, Emergency Medical Services; ICD-10, International Classification of Diseases – 10^th^ revision.

Figure 1 depicts the selection of 9-1-1 EMS events for analysis. From 2018 through 2021, there were 1,079,406 events in which EMS made contact with a pediatric patient and a primary impression was documented. A total of 102,014 (9.5%) of these events had behavioral health provider impressions. In 89% of these events, the clinician documented behavioral health as the primary impression, while the remaining 11% had a behavioral health impression indicated as a secondary impression only.

The annual proportion of eligible pediatric encounters for which clinicians indicated a behavioral health impression ranged from 8.7% to 10.2% (Table 1). Slightly more than one-half of BHE encounters occurred in females (56.2%), and approximately two-thirds occurred in patients aged 14-17 years (68.1%). The majority of incidents (79.4%) occurred in counties characterized as metropolitan areas. The most common scene type was a residence (57.9%), followed by a school (11.6%), or a parking lot, sidewalk, or street/highway (10.2%).

**Table 1.** Demographics of Pediatric Patients Presenting to EMS with BHE, 2018-2021

	Event Year				
Characteristic	2018	2019	2020	2021	Total
**All Pediatric Events**	236,520	279,176	254,234	309,476	1,079,406
**BHE Events**	20,581 (8.7)	25,063 (9.0)	26,008 (10.2)	30,362 (9.8)	102,014 (9.5)
**Sex**					
Female	11,432 (55.5)	13,542 (54.0)	14,600 (56.1)	17,739 (58.4)	57,313 (56.2)
Male	9,056 (44.0)	11,407 (45.5)	11,312 (43.5)	12,423 (40.9)	44,198 (43.3)
Transgender/Non-Binary	23 (0.1)	43 (0.2)	40 (0.2)	112 (0.4)	218 (0.2)
Missing	70 (0.3)	71 (0.3)	56 (0.2)	88 (0.3)	285 (0.3)
**Age (years)**					
0-4	534 (2.6)	669 (2.7)	780 (3.0)	921 (3.0)	2,904 (2.8)
5-10	2,078 (10.1)	2,630 (10.5)	2,246 (8.6)	2,762 (9.1)	9,716 (9.5)
11-13	3,845 (18.7)	4,954 (19.8)	4,940 (19.0)	6,138 (20.2)	19,887 (19.5)
14-17	14,124 (68.6)	16,810 (67.4)	18,042 (69.4)	20,531 (67.6)	69,507 (68.1)
**Urbanicity**^**a**^					
Metro Area (RUCC 1-3)	16,663 (81.0)	20,060 (80.0)	20,593 (79.2)	23,703 (78.1)	81,019 (79.4)
Non-Metro Area (RUCC 4-7)	2,081 (10.1)	2,917 (11.6)	3,464 (13.3)	4,197 (13.8)	12,659 (12.4)
Rural (RUCC 8-9)	190 (0.9)	277 (1.1)	295 (1.1)	405 (1.3)	1,167 (1.1)
Missing	1,647 (8.0)	1,809 (7.2)	1,656 (7.2)	2,057(6.8)	7,169 (7.0)
**Location Found** ^b^					
Residence	11,251 (54.7)	13,339 (53.2)	16,710 (64.2)	17,814 (58.7)	59,114 (57.9)
School	3,219 (15.6)	2,887 (11.5)	1,605 (6.2)	3,168 (10.4)	11,879 (11.6)
Street	1,941 (9.4)	2,525 (10.1)	2,742 (10.5)	3,158 (10.4)	10,366 (10.2)
Health Care Facility	1,233 (6.0)	1,420 (5.7)	1,299 (5.0)	1,669 (5.5)	5,621 (5.5)
Commercial	902 (4.4)	1,184 (4.7)	983 (3.8)	1,467 (4.8)	4,536 (4.4)
Other	685 (3.3)	794 (3.2)	800 (3.1)	835 (2.8)	3,114 (3.1)
Public Area	625 (3.0)	790 (3.2)	609 (2.3)	905 (3.0)	2,929 (2.9)
Recreational Area	243 (1.2)	315 (1.3)	214 (0.8)	336 (1.1)	1,108 (1.1)
Missing	482 (2.3)	1,809 (7.2)	1,046 (4.0)	1,010 (3.3)	3,347 (3.3)

Note: Results presented as n (%).

Abbreviations: BHE, behavioral health emergency; EMS, Emergency Medical Services; RUCC, Rural-Urban Continuum Code.

a Metro Area includes counties located within a metropolitan area that has over 250,000 residents. Non-Metro Area includes urban counties with at least 2,500 residents, that may or may not be adjacent to a metropolitan area. Rural denotes counties that are completely rural or urban with less than 2,500 people in an urban area.

b Location found was categorized using NEMSIS coding as previously described by Rivard, et al (2021)^8^: health care (doctor’s office, hospital, nursing home, other ambulatory care, urgent care); residence (apartment/condo, mobile home, private residence, other private residence); commercial (airport, gym, industrial/construction area, place of business, not otherwise specified (NOS), restaurant/café, store, warehouse); recreation (clubhouse, park, pool, recreational area, NOS, sports area); public area/building; street (parking lot, sidewalk, street/road/highway); and school (daycare, school, school dorm).

Details of how pediatric BHEs presented clinically to EMS are summarized in Table 2 and Table 3. Telecommunicators taking the 9-1-1 calls for these events reported patient complaints related to psychiatric issues, abnormal behavior, a suicide attempt, or overdose/poisoning in 47.4% of the events studied, and reported non-BHE complaints in 34.7%. The most common primary provider impressions documented by on-scene prehospital clinicians were an unspecified mental disorder (43.8%), anxiety or other nonpsychotic mental disorder (21.3%), and mental/behavior disorders related to substance use (19.6%). In 10.8% of events, the clinician indicated a non-BHE primary impression. The primary symptom recorded most often by clinicians was emotional state/behavior, and alcohol/drug exposure was documented as the primary symptom in just 2.3% of events.

**Table 2.** Clinical Presentation to 9-1-1 Telecommunicator for Pediatric BHE Events, 2018-2021

	Event Year				
	2018	2019	2020	2021	Total
**BHE Events (n)**	20,581	25,063	26,008	30,362	102,014
**Complaint Reported by 9-1-1 Telecommunicator**					
Non-BHE Complaint	6,936 (33.7)	8,589 (34.3)	9,041 (34.8)	10,805 (35.6)	35,371 (34.7)
Psychiatric Problem/Abnormal Behavior/Suicide Attempt	6,877 (33.4)	8,571 (34.2)	8,744 (33.6)	9,993 (32.9)	34,185 (33.5)
Overdose/Poisoning/Ingestion	3,048 (14.8)	3,299 (13.2)	3,690 (14.2)	4,150 (13.7)	14,187 (13.9)
Sick Person	1,567 (7.6)	1,988 (7.9)	1,890 (7.3)	2,320 (7.6)	7,765 (7.6)
Unconscious/Fainting/Near-Fainting	1,089 (5.3)	1,313 (5.2)	1,236 (4.8)	1,497 (4.9)	5,135 (5.0)
Unknown Problem/Person Down	589 (2.9)	692 (2.8)	738 (2.8)	769 (2.5)	2,788 (2.7)
Assault	315 (1.5)	460 (1.8)	482 (1.9)	558 (1.8)	1,815 (1.8)
Welfare Check/Well Person Check	111 (0.5)	119 (0.5)	157 (0.6)	220 (0.7)	607 (0.6)
*Missing*	49 (0.2)	32 (0.1)	30 (0.1)	50 (0.2)	161 (0.2)

Note: Results presented as n (%).

Abbreviation: BHE, behavioral health emergency.

**Table 3.** Clinical Presentation to EMS for Pediatric BHE Events, 2018-2021

	Event Year				
	2018	2019	2020	2021	Total
**BHE Events (n)**	20,581	25,063	26,008	30,362	102,014
**EMS Primary Impression**					
Unspecified mental disorder (F99)	9,211 (44.8)	11,134 (44.4)	11,306 (43.5)	13,028 (42.9)	44,679 (43.8)
Anxiety, dissociative, stress-related, somatoform, and other nonpsychotic mental disorders (F40-48)	4,107 (20.0)	5,340 (21.3)	5,495 (21.1)	6,837 (22.5)	21,779 (21.3)
Mental/behavioral disorders due to psychoactive substance use (F10-19)	4,342 (21.1)	5,099 (20.3)	5,155 (19.8)	5,364 (17.7)	19,960 (19.6)
Mood [affective] disorders (F30-39)	627 (3.0)	665 (2.7)	658 (2.5)	764 (2.5)	2,714 (2.7)
Schizophrenia, schizotypal, delusional, and other non-mood psychotic disorders (F20-29)	232 (1.1)	235 (0.9)	275 (1.1)	301 (1.0)	1,043 (1.0)
Behavioral and emotional disorders with onset usually occurring in childhood and adolescence (F90-98)	53 (0.3)	29 (0.1)	75 (0.3)	206 (0.7)	363 (0.4)
Mental disorders due to known physiological conditions (F01-F09)	48 (0.2)	46 (0.2)	28 (0.1)	55 (0.2)	177 (0.2)
Disorders of adult personality and behavior (F60-69)	0 (0.0)	2 (<0.1)	27 (0.1)	38 (0.1)	67 (0.1)
Behavioral syndromes associated with physiological disturbances and physical factors (F50-59)	7 (<0.1)	10 (<0.1)	28 (0.1)	85 (0.3)	130 (0.1)
Pervasive and specific developmental disorders (F80-89)	8 (<0.1)	17 (0.1)	14 (0.1)	18 (0.1)	57 (0.1)
Intellectual disabilities (F70-79)	0 (0.0)	1 (<0.1)	2 (<0.1)	4 (<0.1)	7 (<0.1)
**Non-BHE Primary Impression** ^a^	1,946 (9.5)	2,485 (9.9)	2,945 (11.3)	3,662 (12.1)	11,038 (10.8)
**EMS Primary Symptom,** n (%)					
Emotional State/Behavior	12,088 (55.7)	14,852 (56.2)	15,193 (54.8)	18,197 (55.8)	60,330 (55.6)
Respiratory	899 (3.6)	1,085 (3.4)	1,092 (3.3)	1,245 (3.2)	4,321 (3.4)
Alcohol/Drug Exposure	598 (2.8)	728 (2.8)	561 (2.0)	565 (1.8)	2,452 (2.3)
No Patient Complaint	457 (2.1)	566 (2.1)	707 (2.4)	1228 (3.7)	2,958 (2.6)
Digestive/Abdominal	601 (2.4)	695 (2.2)	699 (2.1)	908 (2.3)	2,903 (2.2)
Injury	636 (2.6)	756 (2.5)	640 (2.0)	463 (1.0)	2,495 (2.0)
Pain	487 (1.7)	554 (1.5)	580 (1.4)	606 (1.3)	2,227 (1.4)
Level of Consciousness	1,712 (6.6)	2,043 (6.6)	2,179 (6.7)	2,439 (6.2)	8,373 (6.5)
Malaise	366 (1.5)	415 (1.4)	391 (1.2)	564 (1.5)	1,736 (1.4)
Musculoskeletal/Nervous	364 (1.2)	499 (1.3)	511(1.1)	636 (1.2)	2,010 (1.2)
Skin	94 (0.4)	86 (0.3)	106 (0.3)	113 (0.3)	399 (0.3)
Illness	148 (0.7)	142 (0.6)	150 (0.5)	147 (0.5)	587 (0.6)
Cardiovascular	76 (0.3)	110 (0.3)	121 (0.4)	147 (0.4)	454 (0.3)
Endocrine	10 (<0.1)	13 (<0.1)	8 (<0.1)	10 (<0.1)	41 (<0.1)
Other	807 (3.4)	943 (3.2)	968 (2.8)	1,065 (2.7)	3,783 (3.0)
Missing	1,238 (5.7)	1,576 (5.8)	2,102 (7.5)	2,029 (6.2)	6,945 (6.3)

Note: Results presented as n (%).

Abbreviations: BHE, behavioral health emergency; EMS, Emergency Medical Services.

a Identified as BHE event based on secondary impression recorded by EMS.

Patients were transported by EMS in 68.9% of events and treatment and/or transport by EMS was refused in 12.5% (Table 4). Across the four years studied, the proportion of events resulting in transport decreased marginally from 69.9% to 67.5%. Clinicians administered sedation medications in 1.9% of encounters and applied physical restraints in 1.7%. The most commonly used medication among those studied was midazolam (1.2%), and naloxone was administered for overdose rescue in 1.5% of events.

**Table 4.** Care Provided by EMS to Pediatric Patients with BHE, 2018-2021

	Event Year				
	2018	2019	2020	2021	Total
**Patient Disposition**					
Treated and Transported	14,389 (69.9)	17,672 (70.5)	17,702 (68.1)	20,491 (67.5)	70,254 (68.9)
Patient Refused Treatment and/or Transport	2,295 (11.2)	2,908 (11.6)	3,529 (13.6)	4,029 (13.3)	12,761 (12.5)
Treated, Transferred Care	2,097 (10.2)	2,338 (9.3)	2,434 (9.4)	2,967 (9.8)	9,836 (9.6)
Treated, Released (per protocol)	936 (4.5)	1,079 (4.3)	1,178 (4.5)	1,590 (5.2)	4,783 (4.7)
Evaluated, No Treatment/Transport Required	760 (3.7)	941 (3.8)	1,031 (4.0)	1,049 (3.5)	3,781 (3.7)
Assists	67 (0.3)	94 (0.4)	112 (0.4)	199 (0.7)	472 (0.5)
Death Pronouncements	10 (<0.1)	9 (<0.1)	11 (<0.1)	10 (<0.1)	40 (<0.1)
**Medications and Procedures**					
Naloxone	303 (1.3)	261 (1.2)	292 (1.8)	462 (1.6)	488 (1.5)
Any Sedation Medication	368 (1.7)	344 (1.9)	473 (2.2)	561 (2.0)	609 (1.9)
Midazolam	211 (1.0)	203 (1.2)	290 (1.3)	329 (1.2)	373 (1.2)
Ketamine	67 (0.3)	61 (0.3)	80 (0.4)	107 (0.4)	117 (0.4)
Lorazepam	59 (0.2)	49 (0.3)	66 (0.3)	76 (0.3)	64 (0.2)
Haloperidol	22 (0.1)	28 (0.1)	25(0.1)	33 (0.1)	41 (0.1)
Diazepam	9 (<0.1)	3 (<0.1)	6 (<0.1)	10 (<0.1)	11 (<0.1)
Ziprasidone	0 (0.0)	0 (0.0)	6 (<0.1)	6 (<0.1)	3 (<0.1)
Physical Restraint	217 (1.6)	330 (1.8)	439 (1.8)	479 (1.6)	496 (1.7)

Note: Results presented as n (%).

Abbreviations: BHE, behavioral health emergency; EMS, Emergency Medical Services.

## Discussion

Understanding the frequency and characteristics of pediatric BHE presentations to EMS can help guide the development of training and protocols to support prehospital clinicians in the management of this growing patient population. Based on this evaluation, an estimated one in ten pediatric EMS encounters occurring in the United States involve a BHE. Just over one-half of EMS-attended pediatric BHE events involve female patients and approximately two-thirds involve patients aged 14 to 17 years. In one-third of the 9-1-1 calls that initiate EMS responses for pediatric BHEs, the telecommunicator records a complaint that is not indicative of a BHE. The current data also suggest that prehospital clinicians very rarely use sedation medications or physical restraint during these encounters, and that 12.5% of pediatric patients seen by EMS for BHEs refuse treatment and/or transport. The uniqueness of EMS usage patterns associated with the coronavirus disease 2019 (COVID-19) pandemic prompted examination of data by calendar year. The general profile of pediatric EMS encounters involving BHEs in terms of the distribution of demographic, clinical, and incident characteristics appeared relatively stable over the study timeframe, with the exception of a noticeable decline in the proportion of BHE responses that occurred in school settings in 2020 that coincides with the period of wide-spread COVID-19 pandemic-related remote learning.^17^

Two previous studies have used national datasets to explore the frequency of EMS encounters involving BHEs in the United States.^7,8^ An analysis of 2018 NEMSIS Version 3 data found that 7.3% of EMS encounters across all patient age groups were related to behavioral health.^8^ Using earlier 2016 NEMSIS Version 2 data, Panchal, et al^7^ reported that the proportion of EMS encounters involving provider impressions of behavioral/psychiatric disorders was 10.6% in adult patients. When they examined children separately, they found that behavioral/psychiatric disorders were the second most prevalent provider impression documented in pediatric EMS events, second only to traumatic injury, and that BHEs accounted for 15.3% of all EMS activations in children. In the current study, a slightly lower prevalence of 9.5% was observed, but this could be explained by differences in both the timeframe examined and the version of NEMSIS data that was used. Provider impressions in the updated Version 3 data reflect the ICD-10 coding system, which is more specific than the ICD-9 codes used in Version 2. Other studies have examined EMS encounters for BHEs in children, albeit not at a national level.^9,10,18^ An estimated 5.7% of all pediatric EMS transports in the state of Florida (USA) from 2011 through 2016 involved BHEs,^10^ and a retrospective study of all EMS encounters that occurred in the city of Baltimore (Maryland USA) concluded that behavioral health problems were a significant cause of repeat use of EMS by patients < 21 years of age.^18^ A recent study from Victoria, Australia also found that mental-health-related presentations accounted for 14% of all pediatric EMS encounters from July 2018 through June 2019,^19^ so commonplace use of EMS for pediatric BHEs may not be unique to the United States. In addition to studies of EMS-specific data, it is estimated that one in four pediatric patients presenting to United States’ EDs with mental health needs arrive by ambulance,^4^ and that 60% of adolescent psychiatrists report recommending to parents that they seek emergency services in the event of a behavioral health crisis.^20^ Taken collectively, these findings indicate that reliance on EMS when seeking care for children experiencing BHEs is considerable.

The significant volume of pediatric BHEs being observed in the prehospital setting is consistent with ED trends and with the larger national crisis of unmet mental health needs in children. National survey data from 2018-2019 indicate one in four youth aged 12-17 reported having received mental health services in the past year,^21^ and self-harm and suicide-related ED visits in pediatric patients increased two- to three-fold from 2007 to 2016.^4,22^ Juxtaposed with rapidly rising patient volumes is a critical national shortage of mental health professionals^23,24^ that leaves many EDs grappling with how to meet the unique needs of pediatric patients experiencing BHEs under traditional models of care. From 2011 through 2015, only 16% of all pediatric patients presenting with mental health diagnoses received care from a mental health professional during their ED encounter, and only 37% of adolescents who arrived in the ED after a suicide attempt or self-harm received an evaluation by a mental health practitioner while in the ED.^4^

As noted in a recent scoping review,^25^ few studies have described the care provided by EMS clinicians during pediatric encounters involving BHEs. In contrast to previously published evidence that roughly 60% of patients who are treated during mental-health-related ED visits receive psychotropic medications,^26^ this study suggests EMS clinicians are unlikely to use pharmacologic treatments for BHEs in the prehospital setting. Administration of sedation medications by EMS clinicians occurred in just 1.9% of encounters in this study, and a previous study of pediatric patients transported by EMS for behavioral health conditions in Florida reported a similar prevalence of 1.3%.^10^ By comparison, 8.0% of pediatric BHE patients treated by EMS in Victoria, Australia receive prehospital parenteral sedation.^19^ With regard to naloxone, a prior study based on EMS data from 2011-2016 found it was administered by EMS clinicians in 0.8% of pediatric behavioral health events.^10^ In the current study, a comparatively higher prevalence of naloxone use of 1.5% was observed, but this may simply reflect the expanding opioid overdose epidemic, increased availability of naloxone, and/or differences in naloxone administration protocols across agencies. Further, children experiencing BHE are unlikely to be physically restrained by EMS clinicians in the United States, as evidenced by the 1.7% and 1.1% prevalence of restraint use reported in this study and a prior study,^10^ respectively. Rivard, et al^8^ examined these same interventions in a national sample comprising largely adult patients (93%) and reported a similar frequency of sedation medication use (1.6%), but found higher frequencies of naloxone (3.0%) and physical restraint use (5.8%). The infrequent use of these interventions, both in adult and pediatric patients, suggests a focus on minimizing sedation and restraint by EMS clinicians in the prehospital management of BHEs.

This study also offers one of the few estimates of the transport rate in pediatric EMS events involving BHEs. Most prior studies from the United States have either excluded non-transported cases from analysis,^9–11^ or have not reported transport rates.^7,18^ Data from this study suggest that 69.8% of EMS activations for pediatric BHEs resulted in transport. This estimate is similar to the overall pediatric transport rate of 69.9% observed in 2019 NEMSIS data,^12^ but is lower than the 88.4% transport rate observed among pediatric EMS activations with a behavioral/psychiatric chief complaint in that dataset. In the recent Australian study by Bourke, et al,^19^ 82% of children with mental health presentations were ultimately transported by EMS. The substantial transport rate in conjunction with the low frequency of interventions in this particular patient subgroup lends itself to exploration of protocols that allow for transport to non-ED/alternative destinations, a model that has been tested with some success in Alameda County, California (USA).^27^

Examination of the 9-1-1 emergency calls that precede paramedic encounters for BHEs suggest that current protocols used by telecommunicators may lack sensitivity for the identification and reporting of BHEs. Non-BHE complaints were documented by telecommunicators in 35% of the pediatric events studied here, and in 48% of EMS events involving BHEs in a similar study of adult patients.^8^ A study conducted in Australia similarly reported that only 47% of adult mental health patients assessed by EMS had their mental health condition identified during the emergency call.^28^ It may be that callers have a tendency to more readily appreciate and report physical symptoms of distress such as altered consciousness, shortness of breath, or injuries resulting from self-harm. Also, current medical dispatching protocols were developed prior to the recent surge in mental illness and direct telecommunicators towards assessments of physical symptoms and signs that have clear care guidelines and are amenable to pre-arrival instructions. Recent work has described a novel training paradigm to optimize recognition of mental illness and educate telecommunicators about mental health crisis care.^29^ Moreover, the International Academies of Emergency Dispatch (Salt Lake City, Utah USA) recently released a revised medical dispatching protocol tailored specifically for behavioral health care scenarios.^30^

## Limitations

This analysis was restricted to 9-1-1 responses and thus did not include patient encounters that occurred during interfacility transfers. The focus of this study was emergent situations, but prehospital clinicians often care for patients with behavioral health conditions when transporting them between medical or other care facilities. Information on patient race is not collected by all EMS agencies in the United States and was missing for 26% of records in this dataset. This precluded useful analysis of the occurrence of pediatric BHEs by race. The geographic representativeness of these data may be limited in that end users of this particular ePCR system are not necessarily evenly distributed across the country. While the Collaborate dataset represents a large (11 million activations annually) and broad (2500+ EMS agencies) sample of EMS activity across the country, these findings can only be considered representative of patients cared for by EMS agencies in the United States that utilize this particular software platform, and sampling error could not be determined due to database inclusion. Lastly, this dataset did not include personal identifiers so the identification of patients who had used EMS more than once during the study timeframe was not possible. Behavioral health conditions may account for a large proportion of repeated use of EMS in pediatric patients,^18^ and thus the absolute number of children receiving EMS care for BHEs is likely less than the counts of encounters presented here.

## Conclusion

These findings suggest BHEs account for a sizeable portion of all 9-1-1 responses that involve children each year, and the majority of cases result in transport of the child. Use of sedation medications and physical restraints by EMS clinicians is exceedingly rare in these events. National EMS data from a variety of sources should continue to be examined to monitor trends in EMS encounters for BHEs in children.
